# Microfungi Associated with *Pteroptyx bearni* (Coleoptera: Lampyridae) Eggs and Larvae from Kawang River, Sabah (Northern Borneo)

**DOI:** 10.3390/insects8030066

**Published:** 2017-07-04

**Authors:** Kevin Foo, Jaya Seelan Sathiya Seelan, Mahadimenakbar M. Dawood

**Affiliations:** Institute for Tropical Biology and Conservation, Universiti Malaysia Sabah, Jalan UMS, 88450 Kota Kinabalu, Sabah, Malaysia; kevinfoo10@yahoo.com

**Keywords:** microfungi, disease profile, congregating fireflies, *Penicillium*, *Trichoderma*

## Abstract

Overlooking the importance of insect disease can have disastrous effects on insect conservation. This study reported the microfungi that infect *Pteroptyx bearni* eggs and larvae during ex-situ rearing project. Two different species of microfungi that infected the firefly’s immature life stages were isolated and identified. *Penicillium citrinum* infected the firefly’s eggs while *Trichoderma harzianum* infected the firefly during the larval stage. Both microfungi species caused absolute mortality once infection was observed; out of 244 individual eggs collected, 75 eggs (32.5%) were infected by *Penicillium citrinum*. All 13 larvae that hatched from the uninfected eggs were infected by *Trichoderma harzianum*. This study was the first to document the infection of *Pteroptyx bearni*’s eggs and larvae by *Penicillium citrinum* and *Trichoderma harzianum*.

## 1. Introduction

Fungi and insects are often closely associated in many habitats, and their interactions ranged from being transient to having obligate associations. Some of the fungi can potentially benefit the insects, while others are detrimental [1]. Several documentations have been done on fungal species that associated fungi with insects (some examples in Table 1). For example, Oliveira et al. [2] studied the fungal diversity found on Olive Moths (*Prays oleae*). Studies done by Ruiz-Najera et al. [3] and Rios-Velasco et al. [4] investigated the entomopathogenic fungi that are associated with fall armyworm larvae (*Spodoptera fragiperda*). A large amount of studies have been done on the fungi associated with Culicidae larvae [5,6,7,8]. *Penicillium citrinum* showed absolute mortality on *Culex quinquefasciatus*’s larvae after bioassay performed with conidia suspension method with concentration of 1 × 10^6^ Conidia per milliliter [8]. *P. citrinum* are known to be associated with mosquito larvae [6], but the infection of fireflies’ egg has not been documented.

Bioluminescence-emitting fireflies have fascinated people across wide geographical regions. The genus, *Pteroptyx*, show very unique behavior, where they congregate in mangrove habitats during the night and flash gregariously to attract the female for mating purposes. The unique ability of the *Pteroptyx* firefly has been exploited by local communities for tourism purposes, such as at Kampung Kuantan in West Malaysia [9]. Congregating fireflies can be found in both Western and Eastern Malaysia. The fireflies (mainly *Pteroptyx tener*) in Kuala Selangor and Sepetang Estuary are currently being utilized as tourist attractions [10,11]. In the state of Sabah, there are several places that are well known for fireflies watching, for example Klias River [12] and Weston [13] where congregating species such as *P. tener*, *P. bearni*, *P. malaccae* and *P. valida* can be found [12,13]. The exponential growth of ecotourism contributes to the livelihood of local communities but poor management of natural resources leads to some irreversible consequences to the ecosystem. Nallakumar [14] reported that the population of *P. tener* in Kampung Kuantan has been declining rapidly as a result of unsustainable activities and pollution. The *Pteroptyx* fireflies are habitat dependent, making the situation worse as it would not disperse to nearby forest with different vegetation structure. If the habitat conservation approach fails to preserve the species in their natural habitat, laboratory breeding and releasing might be the last possible approach to preserve the species. In addition to providing secure genetic reservoirs for *Pteroptyx* fireflies and preparing a large population of fireflies for reintroductions, laboratory breeding projects can also provide detailed documentation of its life history, reproductive biology- and health-related information [15]. This data is also applicable for in-situ population management. To date, there has been very little research done to explore the life history of *Pteroptyx* fireflies. The only available research was done by Ballantyne and Menayah [16], which provided information on the life cycle of *P. valida*. However, information regarding the biology and life cycle of *P. bearni* is still lacking. This knowledge gap also includes the period required for the eggs to hatch, and the clutch sizes.

Microfungi can determine the outcome of the conservation project due to their influences on the surrounding insect communities. Despite their importance to the conservation of firefly, the diversity of microfungi that are associated with fireflies is not well documented. In order to achieve successful laboratory breeding of fireflies, their life-cycle and biology must be well understood. To achieve a better understanding on the biology and life cycle of *P. bearni*, ex-situ rearing programmes were carried out on adult specimens collected from Kawang River. Surprisingly, cotton white hyphae was observed on the firefly’s eggs and larvae during an ex-situ rearing programme, associated with complete mortality of offspring prior to reaching adulthood. Thus, action was taken to identify the exact microbes that infected both the eggs and larvae of *P. bearni* during the ex-situ rearing project.

In this investigation, the Hyphomycetes associated with *P. bearni* eggs and larvae were isolated and identified during the ex-situ rearing project. The data collected from this study will add information to the disease profile of the *Pteroptyx* firefly during laboratory rearing. This documentation can be useful in discovering the methods of avoiding infection during the breeding of this species.

## 2. Materials and Methods

### 2.1. Study Site and Specimen Collection

This study was conducted from January to April 2016 in Kawang River (5°47′30.88″ N, 116°0′16.47″ E), which is located 20 kilometers South of Kota Kinabalu. Adult fireflies of *Pteroptyx bearni* (taxonomic identification was performed by referring to the taxonomic descriptions published by Ballantyne and Lambkin [24]) were collected for the rearing project. A total of 15 mangrove trees with visibly congregating firefly populations were selected for sampling purposes. Sampling was carried out during the night between 7:30 p.m. and 10:30 p.m. with the net sweeping method for two minutes on each mangrove tree where the congregating fireflies were found. Specimens were then placed in the plastic bags until their transfer into disposable petri dishes in the laboratory. The relative humidity in the laboratory was approximately 55.8 ± 5.0%, while the temperature was recorded at 31.0 ± 3.0 °C.

### 2.2. Isolation and Identification of Fungi

The collected fireflies were placed in the disposable petri dishes (with a diameter of 86.5 mm and a height of 14.5 mm), and with moist mud collected from five riverine areas stretching along the Kawang River. The artificial rearing container, a Petri dish filled with 0.5–0.6 mm of non-sterilized, field collected mangrove mud was designed to mimic natural conditions. A total of 40 petri dishes were prepared for the rearing project. Within each disposable petri dish, male and female fireflies were arranged using the ratio of 3 to 2 respectively. Observations were made daily under laminar flow to determine whether eggs were laid, and removing fireflies that showed signs of reduced mobility and were suspected to die soon. The firefly’s eggs were moisturized with 5 mL of sterile (autoclaved) river water daily to avoid dehydration. This was sufficient to keep the firefly eggs from shrinkage, and it would not hinder the mobility of the larvae. Condensed water droplets on the top of the petri dishes were dried every two days with KimWipes (Kimtech Science) under laminar flow. Observation of the fungus on eggs was carried out daily. Once microfungi were observed on the firefly eggs, it was transferred to a petri dish containing Potato Dextrose Agar (PDA) under laminar flow with sterilized needles. Images of the fungi associated with the firefly egg were taken using a Leica Image Analyzer (Model M165C, Leica Biosystems, Nussloch, Germany). Petri dishes were labeled, sealed with Parafilm and incubated at 26 ± 2 °C and examined every two days for one month. The emerged fungal colonies were selected and were transferred into a new petri dish with PDA in order to achieve pure culture.

Larvae which were successfully hatched from the uninfected eggs were kept within the petri dishes with the renewed mangrove mud. The mangrove mud substrates were renewed twice a week. The larvae were fed with living mangrove snails, *Assiminea nitida*, collected from Kawang River, and the snails were rinsed with distilled water before being placed into the rearing petri dishes. The mangrove snail in each petri dish was replaced daily. The larvae were observed daily, once microfungi was observed, it was then cultured into petri dishes containing PDA. Colonies were selected and transferred into a new petri dish with PDA media, the petri dishes were then labeled and sealed with parafilm and incubated at 26 ± 2 °C. Images of the fungi that associate with the firefly larvae were taken by using Leica Image Analyzer (Model M165C).

### 2.3. Microscopic Examination

Cultures were identified microscopically using light microscope (OLYMPUS CX41, Olympus Corporation, Tokyo, Japan) after the fungi were fixed with the scotch tape imprint method [25] and stained with lactophenol cotton blue. Platinum coating on the surface of the microfungi was done by using JEOL auto fine coater (JFC-1600, JEOL Ltd., Tokyo, Japan). The morphology of the *Penicillium citrinum*’s spores were observed with Scanning Electron Microscope (JSM-5610LV, JEOL Ltd., Tokyo, Japan). The identification of fungi from the genus *Penicillium* was based on the taxonomy description published by Houbraken et al. [26]. The identification of *Trichoderma harzianum* was performed by referring to the morphological description published by Kubicek and Harman [27], and the morphological illustrations published by Bissett [28].

## 3. Results

### 3.1. Microfungi Associated with *Pteroptyx bearni* Eggs and Larvae

**Figure 1:**
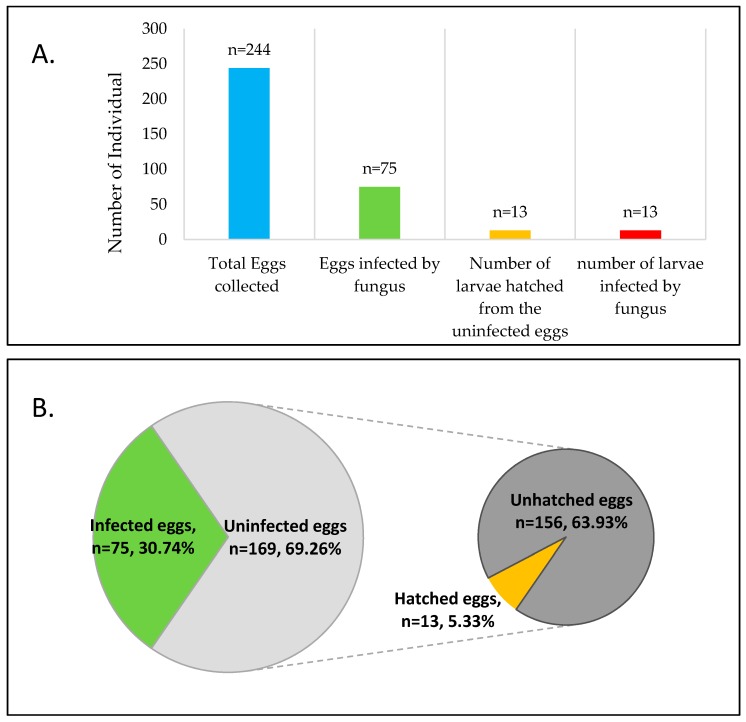
Number of infected eggs and larvae in contrast with the total number of eggs and larvae hatched from the eggs. (**A**) Total of 244 eggs were collected from 40 different matings, representing 120 males and 80 females in individual petri dish in 3:2 ratio. There were 75 eggs infected by fungi and 13 larvae hatched from 169 uninfected eggs; (**B**) The fungus infection rate was 30.74%, while the hatching rate of the uninfected eggs was 5.33%. Number of infected eggs and larvae in contrast with the total number of eggs and larvae hatched from the eggs. (**A**) Total of 244 eggs were collected from 40 different matings, representing 120 males and 80 females in individual petri dish in 3:2 ratio. There were 75 eggs infected by fungi and 13 larvae hatched from 169 uninfected eggs; (**B**) The fungus infection rate was 30.74%, while the hatching rate of the uninfected eggs was 5.33%.

**Figure 2:**
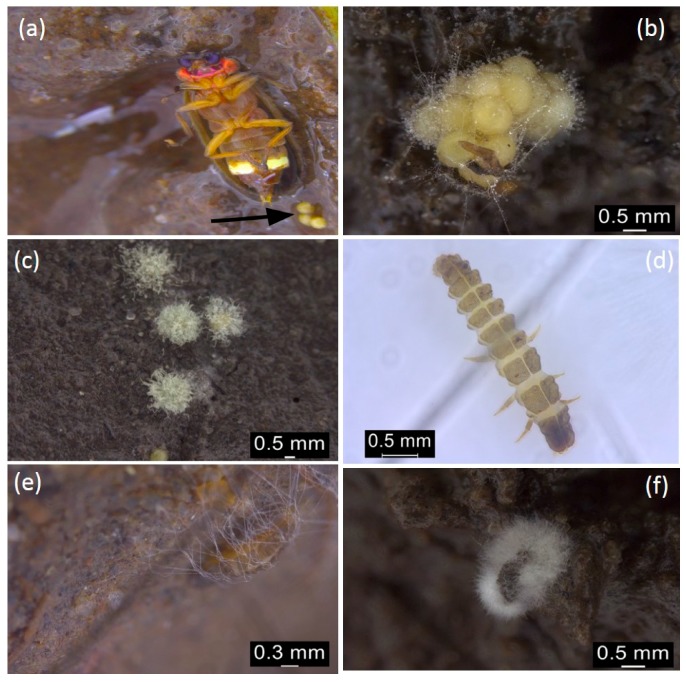
*Pteroptyx bearni*’s eggs: (**a**) Female *Pteroptyx bearni* and its eggs indicated by arrow; (**b**) firefly’s eggs infected by fungus after three days since the first hyphae observed on the surface; (**c**) microfungi infection on the eggs after seven days since the first hyphae was observed. *Pteroptyx bearni*’s larvae: (**d**) *Pteroptyx bearni* larva without infection; (**e**) larvae infected by fungus after three days since the larva become immobile and preparing for ecdysis; (**f**) The larva after ten days since the first hyphae was observed.

Out of 244 *Pteroptyx bearni*’s eggs collected, 75 eggs (Figure 1) were infected by *Penicillium citrinum*. Eggs infected by microfungi showed 100% mortality, where they will not hatch after the presumed period (about one month). The infected eggs shrunk in shape, then turned from light yellowish to brown, and microfungi are observed as outgrowth on the surface of the eggs (Figure 2b,c). Mycelium found on the firefly eggs initially appeared in whitish color and turned into pale greenish in color after one week. All the larvae hatched from uninfected eggs (13 individual hatched from 169 uninfected eggs, shown in Figure 1) were infected by *Trichoderma harzianum* (Figure 4) and eventually led to 100% mortality of the larvae (Figure 1). Fungus infection on the larvae caused the larvae to change in color from light grey to dark brown and the body was observed to shrink in shape (Figure 2e,f).

### 3.2. Penicillium Citrinum Thom

**Figure 3:**
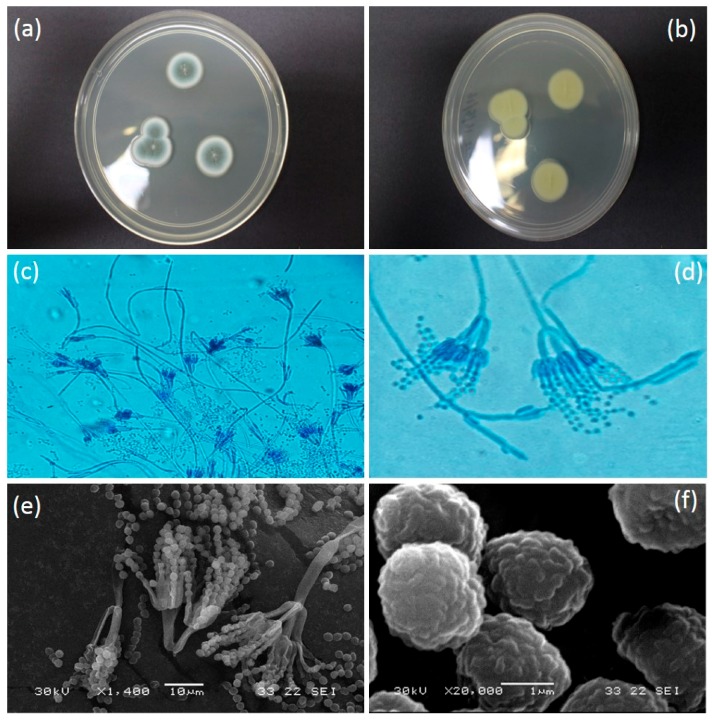
(**a**) Colony surface on PDA media (seven days, 26.8 °C); (**b**) Reverse colony in PDA (seven days, 26.8 °C); (**c**) Hyaline hyphae with smooth-walled conidiophore stripes in 100–300 µm (magnification 400×); (**d**) Conidiophores seen from which extend the metulae and conidia producing phialides; Metulae being substantially longer than the phialides; Metulae are 12–15 µm in length which are found in whorls of 3–5 divergent structures (Magnification 1000×); (**e**) Conidia are globose (round) to sub-globose (with a slightly off-round shape) and present in well-defined chains (Scanning Electron Miscoscope image); (**f**) Conidia (approximately 1.8–2.2 µm measured in diameter) are globose to sub-globose and have finely roughened surface (SEM image).

Colony on PDA media was 21–25 mm in diameter (seven days, 26.8 °C); Colony was grayish turquoise in colour (Figure 3a); Myecelium pure white; Filiform margin with wrinkled surface in crateriform elevation structure; reverse in milky yellowish in colour (Figure 3b). Conidiophore complex and biverticillate conidiophore branching pattern (Figure 3c). Metula presence with bearing terminal verticils of 2 to 5 metulae (mostly observed in 3 metulae) (Figure 3d). Conidiophore with phialides. Conidia spherical (some look slightly ellipsoidal) with rough and irregular surface (Figure 3f). Conidium arising in short to long chains, with 1.5 to 2 µm in diameter (Figure 3e). All colonies derived from eggs had this morphology, consistent with infection by *P. citrinum*.

### 3.3. Trichoderma Harzianum Rifai

**Figure 4:**
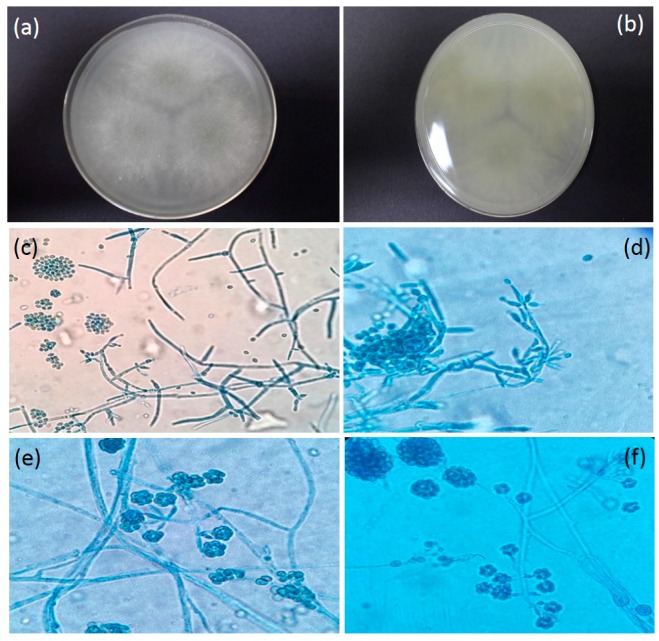
(**a**) Colony surface on PDA media (five days, 26.8 °C); (**b**) Reverse Colony on PDA media (five days, 26.8 °C); (**c**) Septate, Hyaline hyphae with Conidiophores branching at wide angles (near right angle) while some conidia clump together (Magnification 1000×); (**d**) Phialides in flask or ampule shape while some with single conidia are attached to the tips (Magnification 1000×); (**e**) Conidia clustered around tips of the phialides (Magnification 1000×); (**f**) Conidiophores branching at near right angles (Magnification 1000×).

Colony on PDA media was 30–35 mm in diameter (five days, 26.8 °C); Colony is circular as a ring; colony is white as cotton with aerial mycelia; Light grayish in the middle of the colony at day 5 and reached full growth on day 6 on petri dish (Figure 4a); 1–2 concentric rings with dark green conidial production observed on day 7 onwards. The conidia production was denser in the center. The reverse was cotton white in colour and grayish at the middle of the colony (Figure 4b). Hyphae were septated and smooth-walled (Figure 4c). Conidiophore is highly branched and terminates with one or a few phialides that arise directly from the axis near the tip (Figure 4d). Phialides are enlarged in the middle and make it look like a flask (Figure 4e). Conidia exist and will clump together those enlarge in size following the incubation period. Conidia appear in spherical shape (Figure 4f). Only colonies derived from the firefly larvae showed this morphology and was consistent with an infection by *T. harzianum*.

## 4. Discussion

Studies on insect pathology and insect conservation receive a great amount of attention and have made remarkable advances over the last few decades. Unfortunately, both of these principles meet on rare occasions [29]. In order to achieve successful insect conservation, disease and their effects on insect life, especially the developmental stages, must be understood. Despite the ubiquitous existence of microbes, pathogenic and non-pathogenic microbes among insects are not as well documented when compared to other mortality factors such as predators and parasitoids [29]. Fungi associated with insects in various ways, for example, microfungi *Trichoderma* sp. produce lignocellulolictic enzymes that aid in plant’s lignin and cellulose degradation for Oak pinhole borer beetle *Platypus cylindrus* [22]. On the other hand, *Trichoderma* sp. can have catastrophic effect on the larvae of *Simulium goeldii* [30], while several other species of fungi act as the food source for the insect’s larvae (Table 1). From this preliminary study, we documented two species of microfungi that are associated with the firefly *Pteroptyx bearni*’s eggs and larvae, which both caused high mortality during the ex-situ rearing experiment. *Penicillium citrinum* were found to be associated with eggs and *Trichoderma harzianum* were associated with the firefly’s larvae. It is likely that both fungi could be causing high mortality rates for these firefly’s eggs and larvae once association was observed.

Insects are especially vulnerable to microbial infection and predation, as well as the parasitization during the egg development stage due to their immobility, and thus depend on the resources provided by the mother [31,32]. As the eggs commonly accumulate great amounts of nutrients for their development until the hatching of the larvae, they are highly targetable by pathogenic microorganisms [33,34]. Although the insect eggs are immobile, the eggs are not entirely unprotected against attack by pathogens. For example, females of the chrysomelid cucumber beetle *Diabrotica undecimpunctata howardi* endowed their eggs with a chemical (Cucurbitacins) with potential antifungal properties that reduce the pathogenicity of *Metarhizium anisopliae* [33]. In addition, beetles in the families Meloidae and Oedemeridae biosynthesize Cantharidin, which proves to be effective to deter predators and inhibit fungal growth [33]. Although the defensive chemical known as Lucibufagins is utilized by *Photinus* fireflies to deter predators from consuming their eggs [35,36], its antifungal properties were not undocumented.

Hosoe et al. [37] revealed that the chemical excreted by the firefly *Rhagophthalmus ohbai* exhibited antimicrobial activity after being tested through bioassay against fungus isolated from dead fireflies, namely *Aspergillus tubingensis*, *Aspergillus fumigatus*, *Fusarium proliferatum*, and *Trichoderma asperellum.* They suggested that the chemical (1,4-naphthoquinone) plays an important role in conserving their eggs and newly hatched larvae. Unfortunately, the eggs of the firefly species *P. bearni* from this study are highly susceptible to microfungi infection in the laboratory setting, which eventually leads to a 100% mortality rate. The infected eggs were fully covered by microfungi after one week since hyphae were observed and no infected egg managed to hatch any larvae. Females of *P. bearni* do not reveal maternal care towards their eggs compared to *R. ohbai,* which guards its eggs and releases antimicrobial chemicals to prevent pathogenic microorganisms from the soil from infecting its eggs. To the best of our knowledge, there are no documented chemicals that *P. bearni* releases to protect its eggs and newly hatched larvae, as *R. ohbai* does, to maximize the survivorship of its offspring.

In the fireflies’ communities, including the North American *Photinus* fireflies, and Japanese *Luciola* fireflies, whose biology and phenology have been commonly discussed, maternal protection like egg attendance was not documented in both firefly genera. Several types of insects perform egg attendance to remove the microbial infection on the eggs [38,39] which significantly improved hatching rates [40]. Although the firefly parents do exist in the same rearing container with the eggs that were oviposited on the soil, maternal protection such as egg grooming was not observed. During the ex-situ rearing project, several naturally existing organisms were not included in the rearing container. For example, snail communities are widely distributed at the same natural habitats with *Pteroptyx* fireflies. The absence of these snail communities might be linked to the high percentage of *P. citrinum* infection during the ex-situ rearing project due to their natural diet that consists of surface biofilms, which include fungi [41]. The role of snails in removing the surface fungus needs to be clarified through further research. On the other hand, 5 ml of sterile river water was added into the surface of the soil within the rearing container to maintain the moisture content of the mud to avoid desiccation of the firefly eggs. The water availability might otherwise increase the germination rate of fungus that is commonly available in the soil. The data of our own observations and the literature analysis suggest that maintenance of water content within the rearing environment triggered the spore germination and enhanced the mycelium development.

Interestingly, no fungal hyphae were observed on the mangrove mud and the fungal hyphae were only found on both eggs and larvae (as seen in Figure 2c,e). *P. citrinum* are likely to target the glycoprotein and protein source found on the chorionic and exochorion layers of the firefly eggs. The availability of the protein source might be the main triggering agent for the germination of fungal spores. In addition, we suspected that the fungal spores might naturally exist in the mangrove mud and are suppressed by some factors such as solar radiation or liquid precipitation that frequently washes off the fungal spore on the surface of the mud. Laboratory rearing condition could have significantly reduced the reach of UV-radiation that favored the germination of *Penicillium*’s spore, associated with the high water activity; we believed that this is the main reason for fungus infection on firefly eggs. On the other hand, female fireflies are known to breed in the soil of mangrove area where decomposing processes of organic matters are widely occurring. It is interesting to know what mechanisms are applied by *Pteroptyx* fireflies to enhance their offspring fitness as their eggs and larvae are commonly exposed to the widespread soil fungus under natural conditions. From the results, *P. citrinum* was frequently observed from the firefly eggs even in different rearing containers that are physically isolated from other rearing containers. This observation confirmed that the infection of firefly’s eggs by *P. citrinum* is not coincidental because the rearing containers were physically isolated from each other and fungi were observed on the firefly’s eggs from different containers. Several other soil fungi were known to coexist with the *P. citrinum*; what mechanisms are utilized by *P. citrinum* to infect the firefly eggs are still unknown even with the protection of serosa, an extraembryonic epithelium that is capable of defending invading microbial infection and is found in almost all insect species except the higher flies [42].

*Pteroptyx bearni*’s larvae infected by *Trichoderma harzianum* showed 100% mortality, as they could not molt from the first instar to the second instar during their larval stage. The only work in the related literature that reported the existence of *Trichoderma* found on fireflies was that by Hosoe et al. [37]. They isolated four species of microfungi from the dead fireflies *Rhagophthalmus ohbai*, which included the *Trichoderma asperellum*. Only *T. harzianum* was observed to infect the *P. bearni*’s larvae. This observation raises question about why *P. citrinum* that are widely available in the rearing media did not infect the firefly during larvae stage. *T. hazianum* are known to synthesize several types of enzymes (chitinase and protease) and antifungal agents [43]. These antifungal agents could have inhibited the germination and growth of *P. citrinum*, resulting in only one species of microfungi that infect the firefly larvae being identified. From the observation on the fungus growth rate on PDA media, we found that *Trichoderma* expanded their colony’s diameter much more rapidly compared to the *P. citrinum*. We suspect that serosa is capable of inducing the synthesis of antifungal compounds that are able to suppress the spore germination of *Trichoderma* but are not effective in protecting the eggs against *P. citrinum*’s invasion. However, the interaction between *P. citrinum* and *T. harzianum* needs to be investigated in order to verify our assumption about microfungi inter-competition.

There are several improvements that can be done to minimize the fungal infection when rearing the *Pteroptyx* fireflies under laboratory condition. A rearing container with a lights system that emits natural spectrum lights with 2% ultraviolet B and 10% ultraviolet A can be used to suppress germination of the fungal conidia [44]. Moreover, several types of chemical compounds with antifungal properties, including tegosept, methyl paraben, potassium sorbate, and sodium propionate can also be added to the rearing substrate to further minimize fungal growth [45,46].

## 5. Conclusions

This preliminary study reported on the microfungi that are associated with the *Pteroptyx bearni*’s eggs and larvae, providing information on the diseases that result in high mortality of both eggs and larvae during the laboratory rearing program. Anthropogenic activities pose a disastrous effect on the congregating fireflies’ population in several localities in Sabah and Peninsula Malaysia. These firefly populations are currently facing detrimental effects from unsustainable ecotourism practices, habitat destruction, and light pollution. Laboratory breeding and releasing can help to repopulate firefly populations after rehabilitation, but the high infection rate of microfungi towards the fireflies’ eggs in the laboratory setting must be resolved in order to achieve successful breeding. Further research is required to determine the solution to control the growth of *Penicillium citrinum* and *Trichoderma harzianum* on firefly eggs and larvae. The antifungal chemicals mentioned above can be added to the rearing substrate to suppress fungal growth, but experimentation is needed to determine whether there is any side-effect from using these chemicals.

## Figures and Tables

**Table 1:** Fungal diversity and their relationships with insects from literature.

Insect Order (Family)	Species	Microfungi	Host Relationships	Reference
Coleoptera (Leiodidae)	*Zearagytodes maculifer*	*Ganoderma* cf. *applanatum* & *Ganoderma australe*	Fungal as food source for larvae	Kadowaki et al., [17]
Coleoptera (Scolytidae)	*Dendroctonus rufipennis*	*Leptographium abietinum, Ophiostoma ips,* & *Ophiostoma piceae*	Symbiotic	Six & Bentz, [18]
Coleoptera (Silvanidae)	*Ahasverus adrena*	*Aspergillus amstelodam, Penicillium citrinum*, & *Cladosporium* sp.	Fungal as food source for larvae	David et al., [19]
Coleoptera (Tenebrionidae)	*Tribolium castaneum*	*Aspergillus flavus, Aspergillus fumigatus, Penicillium* spp., *Fusarium* spp., & *Rhizopus oryzae*	Symbiotic	Prabha et al., [20]
Coleoptera (Coccinellidae)	*Harmonia axyridis* & *Cheilomenes propinquo*	*Hesperomyces virescens*	Parasitic	Haelewaters et al., [21]
Coleoptera (Platypodidae)	*Platypus cylindrus*	Fungus genus: *Aspergillus, Paecilomyces, Penicillium, Botrytis, Acremonium, Beauveria, Fusarium, Gliocladium, Trichoderma, Raffaelea, Geotrichum, Chaetomium, Scytalidium, Nodulusporium,* & *Streptomyces*	Symbiotic	Henriques et al., [22]
Diptera (Culicidae)	*Aedes aegypti*	*Penicillium citrinum*	Entomopathogenic fungal	Russell et al., [23]
Diptera (Culicidae)	*Culex quinquefasciatus*	*Penicillium citrinum*	Entomopathogenic fungal	Maketon et al., [16]
